# The effect of postdural puncture headache on pre-existing and new-onset headaches after cesarean section: A retrospective study

**DOI:** 10.1016/j.heliyon.2022.e11014

**Published:** 2022-10-10

**Authors:** Mesut Bakır, Şebnem Rumeli, Aynur Özge, Gülçin Gazioğlu Türkyılmaz

**Affiliations:** a Division of Algology, Mersin City Education and Research Hospital, Mersin, Turkey; b Division of Algology, Mersin University Faculty of Medicine, Mersin, Turkey; c Department of Neurology, Mersin University Faculty of Medicine, Mersin, Turkey; d Division of Algology, Bursa City Hospital, Bursa, Turkey

**Keywords:** Obstetric anesthesia, Orthostatic headache, Postdural puncture headache, Spinal anesthesia

## Abstract

**Background:**

Obstetric patients are at higher risk of postdural puncture headache (PDPH) than other age- and sex-matched individuals. The debate over the long-term effects of PDPH continues. In this study, we aimed to assess the development of new-onset headaches or worsening of pre-existing chronic headaches in patients who underwent cesarean section under spinal anesthesia and developed PDPH.

**Methods:**

Forty patients who developed PDPH (Group P) after cesarean section surgery (post-cesarean section–PCS), 80 patients who underwent cesarean section under spinal anesthesia (Group S), and 80 patients who underwent cesarean section under general anesthesia (Group G) were evaluated in the study. Chronic headache and other related symptoms that were present before pregnancy (pre-gestational–PG) and within 12 months after cesarean section were assessed.

**Results:**

Eight of the 40 patients in Group P had a new-onset chronic headache after cesarean surgery, which was significantly higher than the rates in the other groups (p = 0.001). Of the patients whose pre-existing headache worsened during the PCS period, seven were in Group P, and four were in Group S (p = 0.020), while none was in Group G. According to the multiple logistic regression analysis, the risk of worsening headache increased by 1.51-fold for every 1 unit increase in the PG Numerical Rating Scale (NRS).

**Conclusion:**

In conclusion, patients who develop PDPH appear to be at higher risk of developing new-onset headaches or worsening of pre-existing headaches compared with those who do not. We believe that keeping a headache diary for patients who will undergo dural puncture for whatever purpose, and also long-term follow-up of these patients for the risk of chronic headaches may increase awareness of this issue.

## 1 Introduction

The most common complication of neuraxial block is postdural puncture headache (PDPH) [1]. According to the diagnostic criteria of “3rd Edition of International Classification of Headache Disorders (ICHD-3 beta)", dural puncture headache onsets within five days after the incident of dural puncture, cerebrospinal fluid (CSF) leakage in imaging studies and a CSF pressure of less than 60 mmH_2_0 are observed, and it cannot be better characterized by another ICHD-3 diagnosis [2]. Obstetric patients are at higher risk of PDPH than women in their age-matched group [3]. The multicenter Serious Complication Repository (SCORE) study reported that PDPH developed in 0.7% of obstetric patients who underwent neuraxial anesthesia. This rate may vary between 1–10%, depending on the diameter of the spinal needle used. Since PDPH causes limitations in the care of the mother for both herself and her baby, its diagnosis and follow-up should be given serious consideration [4].

Few studies have investigated the risk of developing chronic headaches in patients with PDPH. However, they all examine the relationship between headache and PDPH that develops following undesired dural puncture during epidural interventions rather than spinal [5, 6, 7].

We aim to examine the new-onset headache development and worsening of pre-existing chronic headaches in patients with PDPH who have undergone cesarean section under spinal anesthesia and general anesthesia and investigate the variables associated with this condition.

## 2 Materials and methods

Following the approval by the Mersin University Clinical Research Ethics Committee (References number; 78017789/050.01.04/1280218), anesthesia follow-up forms and algology records of 221 patients who had undergone cesarean section surgery under spinal and general anesthesia between 2014**–**2019 were reviewed. Inclusion criteria for the study were as follows: being between 18**–**40 years old, having undergone cesarean section surgery under spinal or general anesthesia within the past five years, and giving consent for the study. The patients' medical records were evaluated, and their age, comorbidities, date of cesarean section surgery, the type and diameter of the spinal needle used, diagnosis of PDPH, and the treatment given for PDPH (medical treatment, epidural blood patch, etc.) were recorded. Patients who had a cesarean section within the past three months, patients with untreated PDPH, major psychiatric illness, headache attributable to a defined secondary headache cause (ICHD-3: subtype 8–13), patients with BMI >35, and patients who did not issue a study consent as evident from their hospital records were excluded from the study.

### 2.1 Formation of the groups

**Figure 1:**
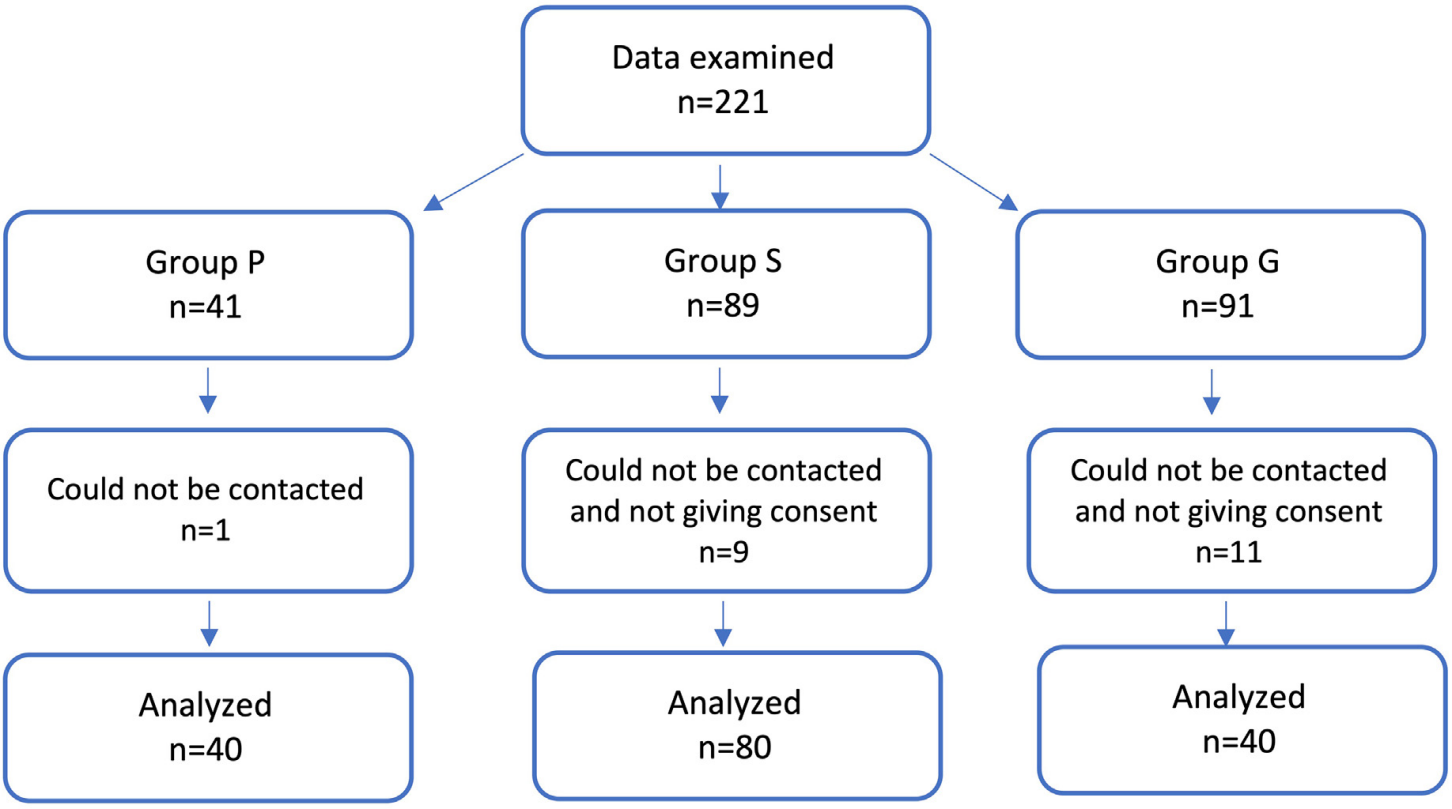
Flow chart.

Algology follow-up files of 40 of 41 patients treated in our algology clinic for PDPH developed during the PCS period within the last five years could be obtained (Group P). In the sample size section of the biostatistics encyclopedia, it is stated that the “asymptotic relative efficiency” value is 0.67 in case of 1 case versus 2 controls [8]. As we discussed with the Biostatistic department, it was agreed that, while the Group P patient number is 40, the number of patients in the other groups should be 80. Patients who underwent cesarean section under spinal anesthesia and did not develop PDPH (Group S) and patients who underwent cesarean section surgery under general anesthesia (Group G) within the same period were randomly reached, and phone calls were continued until the consent of 80 participants for each group was completed. Accordingly, 89 patients for Group S and 91 for Group G were contacted. Twenty-one patients who could not be reached or did not give their consent were excluded from the study (Figure 1).

All patients were asked for the presence of headaches in the PG period, and the frequency, duration, severity, region, presence of aura, nausea-vomiting, photophobia-phonophobia, orthostatic change, and medications were noted. Headaches lasting longer than three months were defined as chronic headaches (PG inquiries were made to make an accurate comparison with the PCS period, as headaches tend to ease during pregnancy). The same questions were asked to the patients for the first 12-month period after cesarean section, and the changes in pre-existing symptoms, as well as the presence of newly onset symptoms lasting longer than three months, were evaluated. All of our questions and evaluations were standardized according to ICHD-3 criteria. All evaluations for the differential-diagnosis of headache were made by one of our authors, headache expert (AO). The answers were recorded in the study information form.

### 2.2 Postdural puncture headache treatment

Per the clinical algorithm used in our unit, rest, hydration, and a combination of paracetamol + caffeine + codeine are used in the first-line treatment for patients with PDPH. However, theophylline is administered for severe headaches (VAS>4). An epidural blood patch is applied for patients with severe headaches or accompanying neurological findings (e.g., tinnitus, abducens paralysis).

### 2.3 Outcomes

Our primary endpoint was the increased incidence of new-onset headaches in patients who developed PDPH. Our secondary endpoint was that dural puncture alone, but not PDPH, could also cause clinical worsening due to dural irritation. In patients with pre-existing headaches in the PG period, a 25% increase in the number of attacks in a month during the PCS period was accepted as clinical worsening.

### 2.4 Statistical analysis

The Statistical Package for Social Sciences version 24 (SPSS v.24) was used for statistical assessments. E-PICOS was also used to stand in good compliance with "Medicres Good Biostatistics Practices”. Descriptive statistics were done for categorical variables, and frequency data were expressed as percentages. The Chi-square test was applied for cross-comparison tables and one-way Anova for variance analysis. Independent group t-test and dependent group t-test were used to compare the mean values between the groups. P < 0.05 was accepted as statistical significance. In patients with clinical worsening, the relationship between different variables was evaluated with multiple logistic regression analysis.

## 3 Results

The mean age of the participants was 31.3 ± 4.5 years in Group P, 30.8 ± 6.1 years in Group S, and 29.7 ± 5.6 years in Group G. The mean ages were similar between the groups (p > 0.05). The records document that a 22-G Quincke-type needle was used for spinal intervention in all patients.

**Figure 2:**
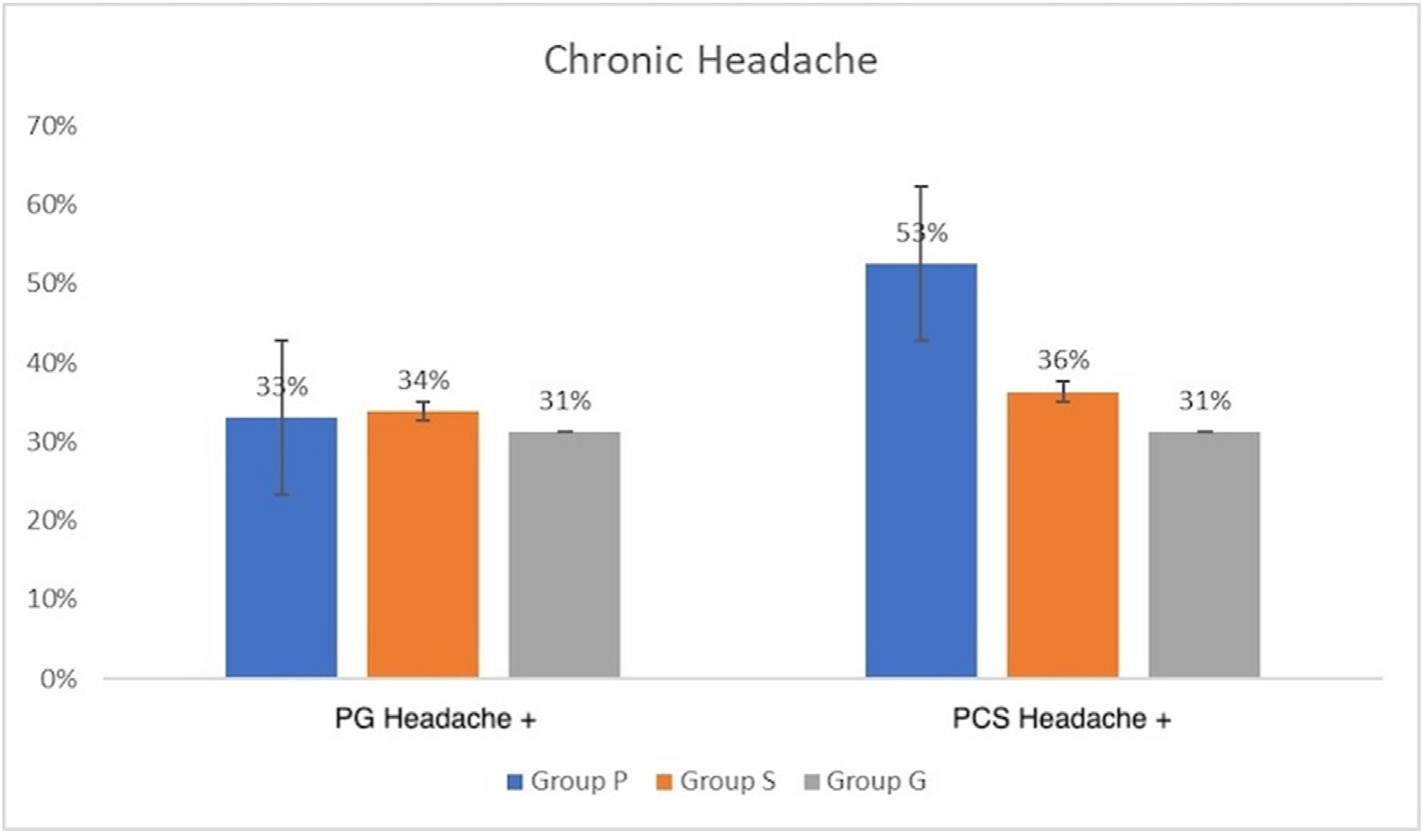
Headache rates of the groups in PG and PCS periods.

Overall, the rate of chronic headache was 32.5% (n = 65) in the PG period and 37.5% (n = 75) in the PCS period. There was no significant difference between the groups regarding pre-existing headache rates in the PG period (p = 0.940). The highest increase in the rate of headache after caesarian section (that is, PCS vs. PG) was in Group P and was significantly different from the other groups (PG period: 32.5%, n = 13; PCS period: 52.5%, n = 21; p = 0.003) (Figure 2). Of the patients with new-onset headache, 80% (n = 8) were in Group P, and 20% (n = 2) were in Group S. No patient with new-onset headache was present in Group G. While this high rate observed in Group P was significantly different from the rates in other groups (p = 0.001), rates in Group S and Group G were statistically similar (p = 0.155). Details of the characteristics of patients with new-onset headache are shown in Table 1.

**Table 1:** Characteristics of the patients with new-onset headache.

No	Age	Group	PDPH Treatment	Aura	Days/Month	Attack Duration^+^	Additional symptom	Location	Allodynia
**1**	33	P	MT	+	8	6	+	Uni^-^	+
**2**	31	P	MT	-	12	4	-	Bi^	-
**3**	39	P	MT	-	8	2	+	Bi	+
**4**	42	P	MT + EBP	-	8	1	-	Bi	-
**5**	30	P	MT + EBP	+	8	6	-	Uni	-
**6**	27	P	MT	+	12	2	-	Uni	+
**7**	23	P	MT	-	4	1	+	Uni	+
**8**	27	P	MT	-	8	48	-	Uni	+
**9**	33	S	None∗	-	8	48	+	Bi	-
**10**	35	S	None∗	-	4	48	+	Uni	-

**MT:** Medical treatment, **EBP:** Epidural blood patch ∗spinal applied patients without PDPH formation ^+^hours ^−^Unilateral ^^^Bilateral.

**Figure 3:**
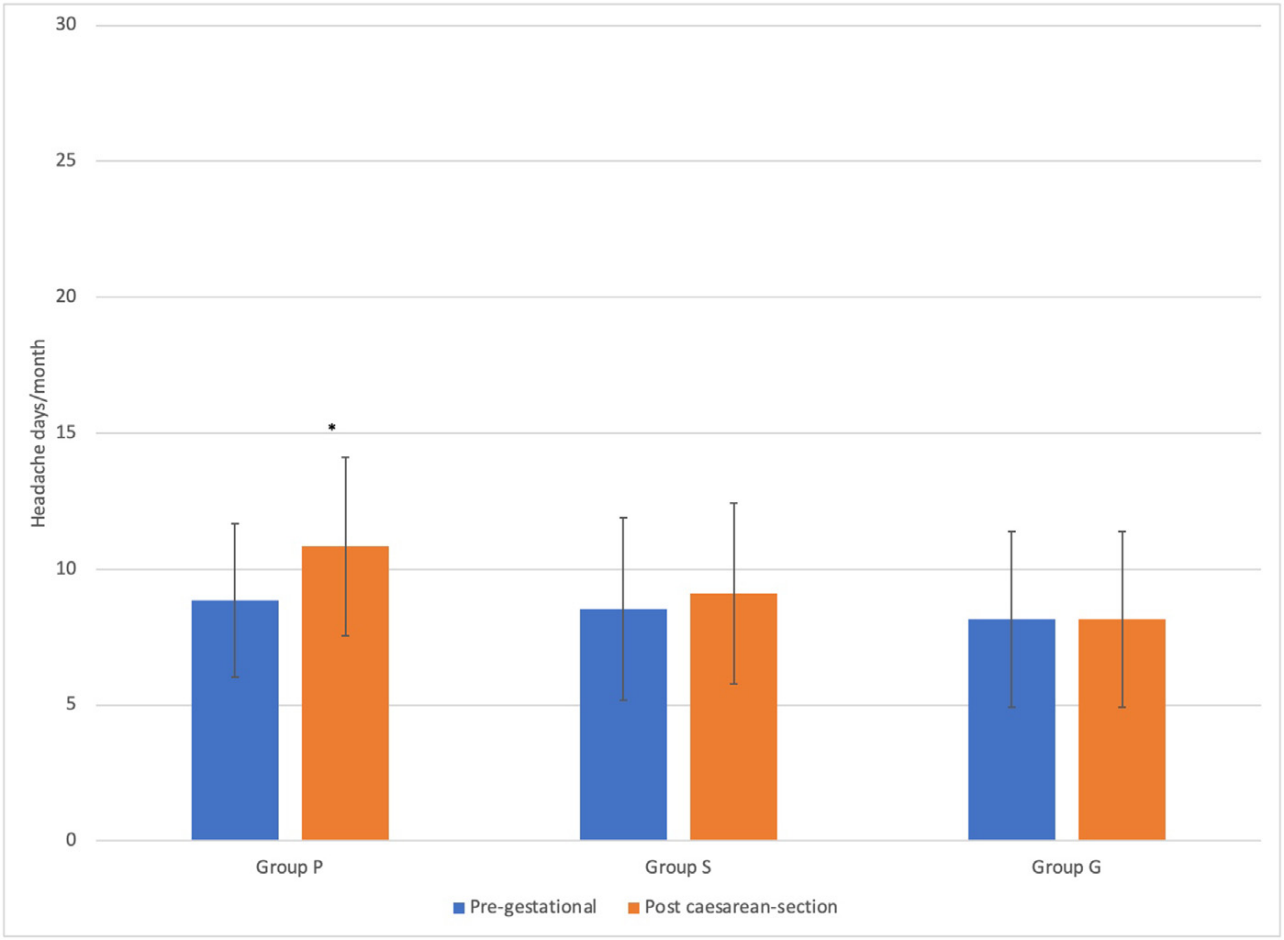
Changes in headache days/month during PG and PCS periods. p = 0.015.

**Figure 4:**
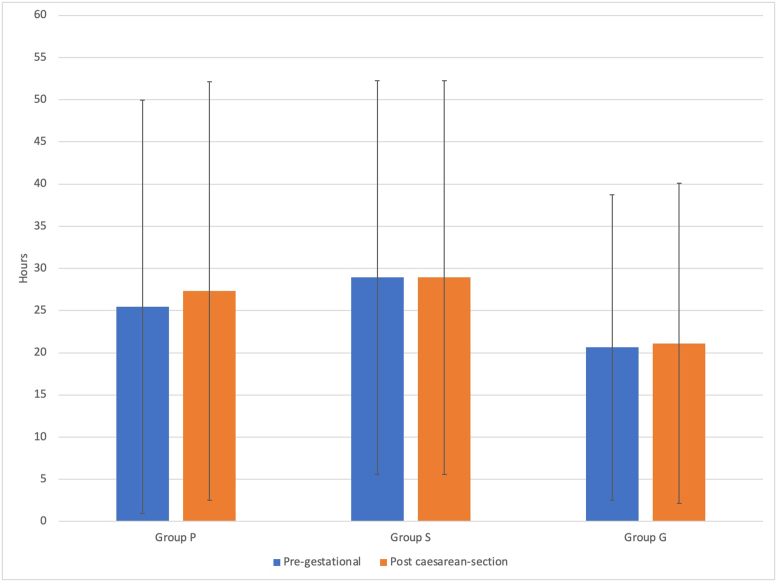
Changes in attack durations of the groups during PG and PCS periods.

When the PG- and PCS periods were compared in terms of the number of painful days/months, a significant increase after caesarian section was found only in Group P (p = 0.015) (new-onset headache was excluded from the analysis to be able to make a comparison between the PG period and the PCS period in terms of the number of painful days/months and number of attacks). There was no significant difference between the groups regarding mean attack durations in the PG and PCS periods (Figures 3 and 4). Headache worsened after cesarean section in 5.5% (n = 11/200) of 65 patients with pre-existing headaches. Of these patients, 63.6% (n = 7) were in Group P, and 36.4% (n = 4) were in Group S. No patient showed worsening of pre-existing headache in Group G. Pre-existing headache worsened in 53.7% (7/13) of the patients in Group P and 14.8% (4/27) of patients in Group S, and the difference between these two groups was statistically significant (p = 0.02).

**Figure 5:**
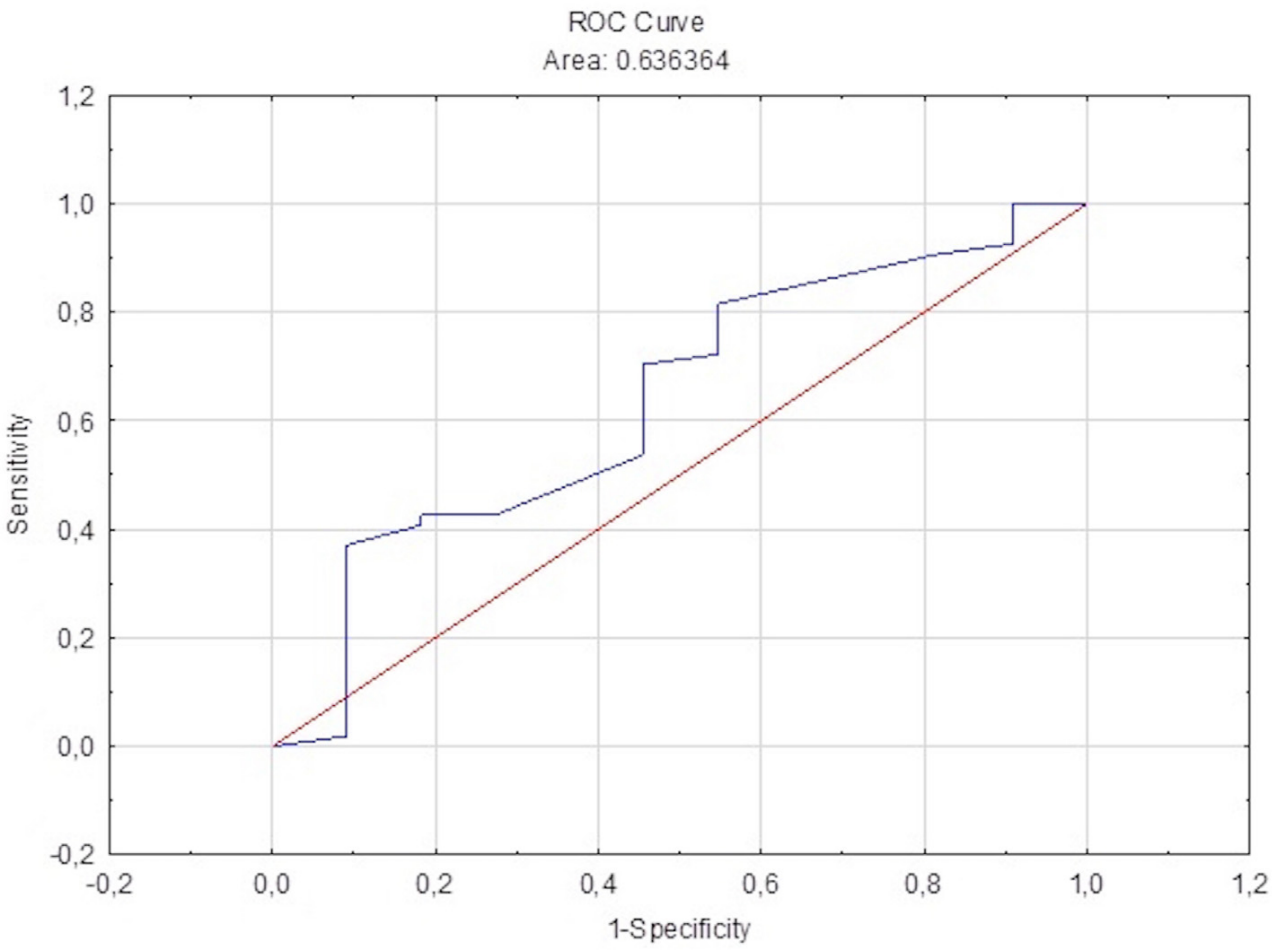
Clinical worsening ROC curve.

We attempted to determine the risk factors that may play a role in clinical worsening with multiple logistic regression analysis. The possible effects of PG NRS scores, headache location, presence of aura, and allodynia on headache worsening after cesarean section surgery were evaluated. We found that for every 1-unit increase in PG NRS scores, the risk of headache worsening increased by 1.51-fold (OR = 1.51 and p < 0.001). However, there was no significant result for headache location, presence of aura, and allodynia. Model fit was tested using the Hosmer-Lemeshow (HL) test (HL = 6.0643 and p = 0.532259; goodness of fit: good). The ROC curve for our model's performance in predicting the worsening of the pre-existing headache is shown in Figure 5.

We evaluated the headache NRS scores of the patients in each group. Intragroup differences between the mean scores measured in the PG vs. PCS periods (Group P: 3.61 ± 0.65 vs. 4.07 ± 0.95; Group S: 4.14 ± 1.10 vs. 4.14 ± 1.10; Group G: 4.24 ± 1.05 vs. 4.24 ± 1.05, respectively) were found to be insignificant (Group P: p = 0.082; Group S: Non-Calculated (NC); Group G: NC).

Headaches were mostly unilateral in both PG and PCS periods in all study groups. The rates of unilateral localization in Group P were 69.2% (n = 9) in the PG period and 66.7% (n = 14) in the PCS period, while the bilateral localization rates were 30.8% (n = 4) in the PG period and 33.3% in the PCS period (n= 7). The rates of unilateral headache localization in Group S were 66.7% (n = 18) in the PG period and 65.5% in the PCS period. In this group, bilateral localization rates were 33.3% (n = 9) in the PG period and 34.5% (n = 10) in the PCS period. Finally, in Group G, unilateral localization rates were 56% (n = 14) in both PG and PCS periods, while bilateral localization rates were 44% (n = 11). When compared in terms of headache location, no significant difference was found between the groups for neither the PG period nor the PCS period (PG: p = 0.700; PCS: p = 0.570).

Rates of accompanying nausea-vomiting and photophobia-phonophobia symptoms did not significantly differ between the PG vs. PCS periods in any of the study groups (for nausea-vomiting: PG period p = 0.521 and PCS period p = 0.391; for photophobia-phonophobia: PG period p = 0.599 and PCS period p = 0.908). In Group P, the presence of aura tended to be more common in patients with pre-existing headaches in the PG period than in the PCS period, albeit without statistical significance (PG period p = 0.487 and p = 0.508 in PCS period). When the PG and PCS periods were compared in terms of the presence of orthostatic symptoms, again, there was no significant difference between the periods in any group (PG period p = 0.830 and PCS period p = 0.215). Three patients in Group P who showed orthostatic symptoms during the PCS period were those with new-onset headaches (Table 2).

**Table 2:** Presence of additional symptoms in the groups in PG and PCS periods.

	Nausea Vomiting		Photophobia Phonophobia		Aura		Orthostatic Headache	
	PG	PCS	PG	PCS	PG	PCS	PG	PCS
***Group P n (%)***	7/13 (53.8)	11/21 (52.4)	7/13 (53.8%)	10/21 (47.6)	6/13 (46.2)	10/21 (47.6)	1 (7.7)	5 (23.8)
***Group S n (%)***	10/27 (37.0)	10/29 (34.5)	10/27 (37.0)	12/29 (41.4)	8/27 (29.6)	10/29 (34.5)	2 (7.4)	2 (6.9)
***Group G n (%)***	9/25 (36.0)	9/25 (36.0)	11/25 (44.0)	11/25 (44.0)	7/25 (28.0)	8/25 (32.0)	3 (12.0)	3 (12.0)

**PG:** Pre-gestational. **PCS:** Post caesarean-section.

After caesarian section, allodynia rates increased from 15% (n = 6) to 42.5% (n = 17) in Group P (p = 0.001), while they remained stable at 15.0% in Group S (n = 12) (p = NC) and at 6.3% (n = 5) (p = NC) in Group G. While there was no significant difference between the groups in terms of allodynia rates in the PG period, the difference between the groups was found to be significant in terms of the rates in the PCS period (p = 0.164 and p = 0.000, respectively). After excluding new-onset headaches, the PG vs. PCS allodynia rates in Group P were found to be statistically similar (p = 0.057).

## 4 Discussion

There is limited data on the association of new-onset or worsening headaches after dural puncture for spinal anesthesia. According to our results, the frequency of pre-existing headaches increases, and new-onset headaches occur in women who have undergone cesarean section under spinal anesthesia and developed PDPH, which indicates its importance.

Variables such as pregnancy, age, and needle diameter are among the variables considered in terms of PDPH development risk after spinal anesthesia [9]. We aimed to increase homogenization among patients regarding age by including only cesarean section patients in our study. In addition, the fact that the study was conducted in a single center helped minimize the variability in the needle diameter and type used.

There are studies investigating long-term headaches in patients who develop PDPH. However, these studies included only patients who had encountered undesired dural intervention during the epidural procedure and eventually developed PDPH rather than spinal. The needle diameter used for epidural intervention is considerably larger than that used for spinal anesthesia; therefore, the dural injury tends to be more prominent in these cases. Ranganathan et al. investigated the long-term effects of undesired dural puncture during epidural intervention. They found that the incidence of chronic headache in these patients was significantly higher than in the control group (34.9% vs. 2.2%) [10]. Similarly, Orbach-Zinger et al. reported that the incidence of long-term headache was higher in patients who developed PDPH after epidural anesthesia compared to the control group (42/129 (32.6%) vs. 42/276 (15.2%)) [11]. In another prospective study, patients who had an accidental dural puncture during epidural intervention were followed up for 18 months. It was reported that 30% (n = 12/45) of these cases had a persistent headache, of whom six developed a new-onset headache, and six experienced worsening of their pre-existing headaches [12]. However, these reports do not include a comparison with cases undergoing spinal intervention. In our study, the rate of patients who developed new-onset headaches was 20% (8/40) in the PDPH group, compared to only 2.5% (2/80) in the spinal group. The fact that we did not detect any new-onset headache or worsening of the pre-existing headache in the general anesthesia group suggests that PDPH poses a higher risk. At the same time, we failed to detect new-onset headaches in the spinal intervention group suggesting that dural irritation may also be another risk factor.

The risk of developing PDPH after spinal intervention in patients with primary headaches has been investigated. Kuntz et al. reported that patients with primary headaches are more prone to develop PDPH [13]. In our study, the incidence of headache in the PG period was similar in all groups. Previous studies have strongly demonstrated that fluctuations in estrogen levels increase headache frequency during the postpartum period [14]. Following pregnancy, where hemodynamic and hematological changes are evident, the return to normal physiology occurs during the puerperal period. This is found to be closely related to headaches [15, 16]. Our results reveal headache rates similar to the previous studies in both PCS and PG periods, both in the spinal group without PDPH and in the general anesthesia group, which can be attributed to the fact that our patients' post-cesarean period headache inquiries were made after three months when the hormonal fluctuation decreases. However, the significant increase in the frequency of chronic headaches in patients with PDPH is important because it suggests that the effects of PDPH may persist in the long term.

Although the scores in the PG and PCS periods were similar in terms of NRS values in the groups, the relationship between the increase in NRS value and clinical worsening was remarkable in the multiple logistic regression evaluation. To the best of our knowledge, there is no study in the literature to compare NRS values.

The similarities between the characteristics of chronic headache in patients with PDPH and other primary headache symptoms (aura, phonophobia-photophobia, nausea-vomiting, aura, allodynia, orthostatic symptoms) have not been studied before. It has been shown in numerous studies that migraine-type headache is mostly unilateral (60%), and this feature is already established in the diagnostic algorithms. In our study, the aura rates in patients who did not develop PDPH and patients who received general anesthesia agree with the literature but tend to be higher in the PDPH group. However, aura rates did not significantly differ between the study groups, and including the patients with new-onset headaches does not change this result. This indicates that there is no association between the development of PDPH and the presence of aura and additional symptoms. PDPH is characterized by orthostatic headache. However, the mechanisms underlying the symptoms of conditions such as migraine, chronic pain, and postural orthostatic tachycardia syndrome (POTS) show similarities to some extent [17]. POTS patients very often (94%) have orthostatic symptoms [18]. The orthostatic symptom incidence in our patients was similarly low in all groups. However, it was remarkable that orthostatic symptoms tended to persist in the long term in all three patients that developed new-onset headaches. Allodynia is defined as stimuli that cause sensory discomfort or pain that would not have any effect under normal conditions. In functional imaging studies, it has been shown that individuals with migraine are hypersensitive to sensory stimuli even between the attacks [19]. Allodynia can be an additional symptom in migraine patients [20]. Dodick et al. evaluated 15133 migraine patients in their study, and allodynia frequency was 39.9% [21]. In our study, while PG allodynia rates were similar between the groups, there was a significant increase in post-cesarian allodynia rates in the PDPH group. The statistical significance disappears when patients with new-onset headaches are excluded from the analysis, suggesting that allodynia rates are higher in patients whose headaches developed after PDPH than in the other patient groups. Considering all the symptoms, it is evident that the pain characteristics in those who developed chronic headaches in our study are more similar to those of migraine-type headaches.

The main strength of this study is that spinal needles of the same type and diameter were used in all of the included patients. This allowed the analysis of a homogeneous patient group, despite the retrospective design. Another strength is that spinal anesthesia patients exposed to dural irritation (lumbar puncture) were included in the control group, as well as patients who had never been exposed to dural irritation (general anesthesia group).

### 4.1 Limitations

Although the number of patients included in the study was 200, statistical analyses were sometimes suboptimal because the number of patients with new-onset headaches detected during the PCS period was only ten. Another limitation is that our patients do not keep a headache diary.

## 5 Conclusion

In conclusion, patients who develop PDPH seem to be at higher risk of developing new-onset headaches or worsening of pre-existing headaches than those who do not. However, we believe that more accurate interpretations of whether dural irritation that develops during intervention with spinal needles causes new-onset headaches or worsening of pre-existing headaches can be made after further studies with larger patient groups. However, we believe that these results should be taken into consideration when informing patients with primary headaches who will undergo spinal anesthesia.

## Declarations

### Author contribution statement

Mesut Bakır: Conceived and designed the experiments; Performed the experiments; Analyzed and interpreted the data; Contributed reagents, materials, analysis tools or data; Wrote the paper.

Şebnem Rumeli: Conceived and designed the experiments; Analyzed and interpreted the data; Contributed reagents, materials, analysis tools or data; Wrote the paper.

Aynur Özge: Conceived and designed the experiments; Analyzed and interpreted the data; Wrote the paper.

Gülçin Gazioğlu Türkyılmaz: Performed the experiments; Contributed reagents, materials, analysis tools or data; Wrote the paper.

### Funding statement

This research did not receive any specific grant from funding agencies in the public, commercial, or not-for-profit sectors.

### Data availability statement

Data included in article/supp. material/referenced in article.

### Declaration of interest’s statement

The authors declare no conflict of interest.

### Additional information

No additional information is available for this paper.

## References

[1] Buddeberg B.S., Bandschapp O., Girard T. (2019). Post-dural puncture headache. Minerva Anestesiol..

[2] (2013). The international classification of headache Disorders, 3rd edition (beta version). Cephalalgia.

[3] Uluer M.S., Sargin M., Akin F., Uluer E., Sahin O. (2019). A randomized study to evaluate post-dural puncture headache after cesarean section: comparison with median and paramedian approaches. Niger. J. Clin. Pract..

[4] D'Angelo R., Smiley R.M., Riley E.T., Segal S. (2014). Serious complications related to obstetric anesthesia: the serious complication repository project of the Society for Obstetric Anesthesia and Perinatology. Anesthesiology.

[5] Webb C.A., Weyker P.D., Zhang L. (2012). Unintentional dural puncture with a Tuohy needle increases risk of chronic headache. Anesth. Analg..

[6] Ljubisavljevic S., Zidverc Trajkovic J. (2020). Postdural puncture headache leads to clinical worsening of pre-existing chronic headache. J. Clin. Neurosci..

[7] Jeskins G.D., Moore P.A., Cooper G.M., Lewis M. (2001). Long-term morbidity following dural puncture in an obstetric population. Int. J. Obstet. Anesth..

[8] Wacholder S., Hartge P. (2005).

[9] Amorim J.A., Gomes de Barros M.V., Valença M.M. (2012). Post-dural (post-lumbar) puncture headache: risk factors and clinical features. Cephalalgia.

[10] Ranganathan P., Golfeiz C., Phelps A.L. (2015). Chronic headache and backache are long-term sequelae of unintentional dural puncture in the obstetric population. J. Clin. Anesth..

[11] Orbach-Zinger S., Eidelman L.A., Livne M.Y. (2021). Long-term psychological and physical outcomes of women after postdural puncture headache: a retrospective, cohort study. Eur. J. Anaesthesiol..

[12] Gauthama P., Kelkar A., Basar S.M.A., Niraj G. (2019). Incidence of persistent headache at 18 Months following accidental dural puncture in the obstetric population: a prospective service evaluation in 45 patients. Headache.

[13] Kuntz K.M., Kokmen E., Stevens J.C., Miller P., Offord K.P., Ho M.M. (1992). Post-lumbar puncture headaches: experience in 501 consecutive procedures. Neurology.

[14] Mattsson P. (2003). Hormonal factors in migraine: a population-based study of women aged 40 to 74 years. Headache.

[15] Sader E., Rayhill M. (2018). Headache in pregnancy, the puerperium, and menopause. Semin. Neurol..

[16] Klein A.M., Loder E. (2010). Postpartum headache. Int. J. Obstet. Anesth..

[17] Cook G.A., Sandroni P. (2018). Management of headache and chronic pain in POTS. Auton. Neurosci..

[18] Shaw B.H., Stiles L.E., Bourne K. (2019). The face of postural tachycardia syndrome–insights from a large cross-sectional online community-based survey. J. Intern. Med..

[19] Schwedt T.J., Chiang C.C., Chong C.D., Dodick D.W. (2015). Functional MRI of migraine. The lancet. Neurology.

[20] Burstein R., Noseda R., Borsook D. (2015). Migraine: multiple processes, complex pathophysiology. J. Neurosci..

[21] Dodick D.W., Reed M.L., Fanning K.M. (2019). Predictors of allodynia in persons with migraine: results from the migraine in America symptoms and treatment (MAST) study. Cephalalgia.

